# Effects of Stromal Cell‐Derived Factor 1 on Osteoblast Activity During Orthodontic Tooth Movement

**DOI:** 10.1002/cre2.70425

**Published:** 2026-08-13

**Authors:** Yoshiyuki Hamada, Yuji Ishida, Albert Chun‐Shuo Huang, Kenta Hamahara, Duangtawan Rintanalert, Kai Li, Kasumi Hatano‐Sato, Jun Hosomichi, Risa Usumi‐Fujita, Takashi Ono

**Affiliations:** ^1^ Department of Orthodontic Science, Graduate School of Medical and Dental Sciences Institute of Science Tokyo Japan; ^2^ Mirai Dental Clinic New Taipei City Taiwan; ^3^ Department of Orthodontics Chulalongkorn University Thailand; ^4^ Center of Excellence in Precision Medicine and Digital Health, Department of Physiology, Faculty of Dentistry Chulalongkorn University Bangkok Thailand

**Keywords:** chemokine receptor type 4, orthodontic tooth movement, osteoblast, stromal cell‐derived factor 1

## Abstract

**Objective:**

This study aimed to clarify the relationship between stromal cell‑derived factor 1 (SDF‑1) and osteoblast‐related responses in periodontal ligament (PDL) during orthodontic tooth movement (OTM) after tooth extraction, based on the hypothesis that local inhibition of SDF‑1/C‑X‑C chemokine receptor type 4 (CXCR4) signaling with the CXCR4 antagonist AMD3100 would attenuate osteoblast differentiation and alveolar bone remodeling during OTM.

**Methods:**

Thirty‐six 6‐week‐old Wistar/Sprague–Dawley male rats were randomly divided into control and experimental groups. After the extraction of the maxillary left first molar, the maxillary left second molar (M2) was moved mesially with a 10‐gf force following the local injection of phosphate‐buffered saline or AMD3100 into the buccal and palatal gingiva adjacent to the M2 in the control and experimental group, respectively. Micro‐computed tomography was performed to assess tooth movement and alveolar bone changes. Histological and immunohistochemical analyses were performed to evaluate periodontal tissue morphology and the percentages of SDF‐1‐, CXCR4‐, RUNX2‐, and BGLAP‐positive cells on the tension side of the PDL. Quantitative reverse transcription PCR was used to assess expression levels of SDF‐1, CXCR4, RUNX2, and BGLAP.

**Results:**

OTM distance and mesial tilt angle were significantly reduced in the experimental group. RT‐PCR showed that SDF‐1, CXCR4, and RUNX2 expression levels were significantly downregulated from Day 1 through Day 7, whereas BGLAP expression was significantly decreased only on Day 1. Immunohistochemical analysis of the tension side of the PDL demonstrated significantly reduced SDF‐1‐ and CXCR4‐positive cells on Day 3 and RUNX2‐positive cells on Day 7 in the experimental group. In contrast, the BGLAP‐positive cells did not differ significantly at any time point.

**Conclusion:**

Local inhibition of SDF‐1/CXCR4 signaling reduced OTM and altered early alveolar bone remodeling after tooth extraction. The combined gene expression and immunohistochemical findings suggest that the SDF‐1/CXCR4 axis may contribute to osteoblast differentiation, particularly through RUNX2‐related responses during the early phase of OTM.

**Trial Registration:**

Not applicable.

## 1 Introduction

Orthodontic tooth movement (OTM) is a biological process involving the periodontal tissues, including remodeling of the alveolar bone and periodontal ligament (PDL) in response to applied orthodontic forces. Bone maintains homeostasis through a delicate balance of resorption by osteoclasts and formation by osteoblasts (Huang et al. 2014). A previous study reported that osteocytes regulate osteoblast and osteoclast differentiation in response to mechanical loading in bone (Matsumoto et al. 2013). Collectively, bone remodeling is finely organized by the interactions of osteoclasts, osteoblasts, and osteocytes.

Osteoclast formation is regulated by various cytokines, including the receptor activator of nuclear factor kappa B ligand (RANKL), interleukin (IL)−6, tumor necrosis factor (TNF)α, and transforming growth factor (TGF)‐β (Yasuda et al. 1998; Gao et al. 1998; Fox et al. 2000). Conversely, osteoblasts are derived from mesenchymal stem cells via stimulation with various hormones and cytokines. Stem cells differentiate into pre‐osteoblasts and osteoblasts, and mature osteoblasts eventually transform into osteocytes. Runt‐related transcription factor 2 (RUNX2) and Osterix are essential transcription factors for osteoblast differentiation (Adhami et al. 2014; Nishimura et al. 2012).

At sites of bone resorption caused by osteoclastic activity, mature osteoblasts generate new bone and secrete bone matrix proteins, such as type 1 collagen, osteopontin (OPN), and osteocalcin (OCN) (Garlet et al. 2007) to promote extracellular matrix calcification. Moreover, these cells secrete cytokines, such as TGF‐β, insulin‐like growth factor 1 (Xian et al. 2012) fibroblast growth factor 2, and bone morphogenetic proteins (BMP) (Nishimura et al. 2012; Xian et al. 2012; Yu et al. 2016). During osteoblast differentiation, protein expression patterns change, with increased expression of alkaline phosphatase and OPN as markers of early to mid‐stage osteoblast differentiation (Nugraha et al. 2018; Ram et al. 2015), while OCN and bone sialoprotein are induced as markers of late differentiation (Garlet et al. 2007; Ogata 2008).

Chemokines involved in the migration of osteoblasts and osteoclast precursors, as well as in the formation and maintenance of osteocytes, have also been identified (Wright and Friedland 2004; Bendre et al. 2003). Chemokines are a group of low‑molecular‑weight cytokines that primarily exert migratory effects on cells bearing chemokine receptors, such as hematopoietic stem cells and leukocytes. A previous study showed that stromal cell‐derived factor‐1 (SDF‐1), also known as C‐X‐C motif chemokine ligand 12 (CXCL12), is upregulated in damaged bone tissue during healing and that the SDF‑1/CXCR4 axis directs bone‑marrow stem cells (BMSCs) from the bone marrow, blood vessels, and periosteum to the injured tissue (Yellowley 2013). Our research group has shown that inhibiting SDF‐1/CXCR4 signaling reduces OTM and osteoclast accumulation on the root compression side (Hatano et al. 2018). In addition, local administration of anti‐SDF‐1 antibodies to directly inhibit SDF‐1 signaling has been shown to reduce the inflammatory response of the PDL associated with tooth movement, thereby inhibiting tooth movement.

Collectively, current research studies suggest that SDF‑1 contributes to the accumulation of BMSCs on the compression side of the PDL and to the recruitment of osteoclasts within periodontal tissues during OTM. On the tension side of the PDL, SDF‑1 is also expressed because it binds to the promoter region of RUNX2 and directly up‑regulates RUNX2 transcription. SDF‑1 is presumed to participate in bone formation on this side during the application of orthodontic forces. Nevertheless, the relationship between SDF‑1 and osteoblast activity during OTM remains unclear. We hypothesize that SDF‑1 plays a pivotal role in osteoblastic activity and bone formation within the PDL during OTM. Accordingly, this study aimed to determine whether inhibition of SDF‑1/CXCR4 signaling attenuates osteoblastic activity and bone formation during OTM in the PDL.

## 2 Materials and Methods

### 2.1 Experimental Animals

All animal experiments were approved by the Institutional Animal Care and Use Committee of the Institute of Science Tokyo (Approval No. 2021115C). This study adhered to the ARRIVE (Animal Research: Reporting of In Vivo Experiments) guidelines. Six‐week‐old male Wistar Sprague–Dawley (SD) rats (Japan SLC, Shizuoka, Japan; *n* = 36) were used in this study. The rats were randomly divided into two groups. The control group underwent extraction of the upper left first molar (M1), followed by injection of phosphate‐buffered saline (PBS) into the buccal and palatal gingiva adjacent to M2 and OTM of the upper left second molar (M2). The experimental group underwent the same M1 extraction, followed by injection of the selective CXCR4 antagonist AMD3100 (AdooQ Bioscience, CA, USA) in the same manner as the control group and M2 OTM. All rats were acclimatized for 1 week and then allowed to consume powdered feed and water *ad libitum* in a controlled environment during the experiment (22 ± 1°C, standard 12‐h light–dark cycle).

### 2.2 Tooth Extraction and OTM Model

Systemic anesthesia was induced by subcutaneous injection of a mixture containing medetomidine (0.3 mg/kg), midazolam (4 mg/kg), and butorphanol (5 mg/kg). The upper left first molar (M1) was then extracted. A 10‐gf nickel‐titanium closing coil spring (Tomy International, Tokyo, Japan) was attached to the upper incisors with a stainless ligature wire (Tomy International) and fixed in place using light‐curing composite resin (GC, Tokyo, Japan) to move the upper left second molar (M2) mesially. After placement of the coil spring, the experimental group received local injections of AMD3100 (solution concentration 1,000 µM, delivering 0.25 mg/kg) into the buccal and palatal gingiva adjacent to M2 (Okada et al. 2016). The control group was injected with an equivalent volume of phosphate‐buffered saline (PBS) using the same protocol (Figure S1). Following all procedures, recovery from anesthesia was facilitated by subcutaneous administration of atipamezole hydrochloride (0.75 mg/kg; a medetomidine antagonist), buprenorphine hydrochloride (0.03 mg/kg), and penicillin G potassium (200,000 U).

### 2.3 Micro‐Computed Tomography (CT) Analysis

At the time of the micro‐CT examination, the head and abdomen were fixed in the supine position under systemic anesthesia. Micro‐CT of the head was performed using an R_mCT2 SPMD (Rigaku, Tokyo, Japan). The micro‐CT settings were 90 kV, 160 mA, and a 30‐mm field of view. In the micro‐CT image, the amount of OTM was defined as the mesiodistal distance from a reference line, which passes through the interdental space between M2 and M3 on the contralateral non‐moved control side and perpendicular to the mid‐palatal suture, to the distal surface of M2 on the experimental side (Figure S2). Moreover, the change in the angle between the M2 proximal root and occlusal plane before and after OTM on the sagittal plane was measured. At 1, 3, and 7 days after OTM, the rats were euthanized by cardiac puncture under deep anesthesia, and peripheral blood was collected for subsequent enzyme‐linked immunosorbent assay (ELISA) analysis. The maxillae were excised immediately. After sectioning, all samples were scanned using a desktop X‐ray micro‐CT system (SMX‐100CT, Shimadzu, Kyoto, Japan). The microstructure of the alveolar bone was analyzed using three‐dimensional (3D) image analysis software (TRI/3D‐BON, Ratoc System Engineering, Tokyo, Japan), following the software's instructions. The alveolar bone in the maxillary M2 was chosen as the region of interest (ROI) for bone analysis (Figure S3).

### 2.4 Histological and Immunohistochemical Preparation

Following micro‐CT, the maxillae were immediately resected. Samples were fixed in paraformaldehyde (4%, pH 7.4; Wako Pure Chemicals, Osaka, Japan) for 24 h at room temperature, and subsequently stored in PBS (pH 7.4; Wako Pure Chemicals) at 4°C. Fixed maxillae were decalcified with 10% ethylenediaminetetraacetic acid (pH 7.2) for 30 days at 4°C and embedded in paraffin using a conventional method. Specimens were sliced into 5‐μm‐thick sections using a microtome (HistoCore AUTOCUT, Leica Biosystems GmbH, Nussloch, Germany), and the stained sections were scanned using a slide scanner (Slideview VS200, Olympus, Tokyo, Japan) and analyzed for histological evaluation. Furthermore, using horizontal sections, we measured the width of the PDL on both the compression and tension sides of the mesial palatal root of M2.

For immunohistochemical analysis, deparaffinized sections were subjected to antigen retrieval and endogenous peroxidase blocking. After blocking nonspecific binding, the sections were incubated overnight at 4°C with primary antibodies against SDF‐1 (Novus Biologicals, NBP2‐12221), CXCR4 (Bioss, bs‐1011R), RUNX2 (Abcam, ab23981), and BGLAP/Osteocalcin (Abcam, ab93876), each diluted 1:200. The sections were subsequently incubated with appropriate secondary antibodies, and immunoreactivity was visualized using 3,3′‐diaminobenzidine (DAB). The sections were counterstained with hematoxylin and scanned using the VS200 system.

Quantitative analysis of immunohistochemical staining was performed using ImageJ software (National Institutes of Health, USA). Images were acquired at ×400 magnification from the distal surface of the distopalatal root of the maxillary second molar (M2), including the PDL and adjacent alveolar bone. Three non‐overlapping regions of interest (ROIs; 80 μm × 80 μm each) were selected for each section. The percentage of immunopositive cells was calculated as the number of positively stained cells divided by the total number of cells within the ROIs. The mean value of the three ROIs was used for statistical analysis.

### 2.5 SDF‐1 Concentration in Peripheral Blood

Peripheral blood was collected via cardiocentesis 1, 3, and 7 days after OTM. Mononuclear cells and serum were collected separately using a density‐gradient cell separation tube (BD Vacutainer CPT cell preparation tube with sodium citrate; BD Biosciences). The concentration of SDF‐1 in the serum was examined using a mouse CXCL12/SDF‐1 alpha Quantikine ELISA kit (MCX120; R&D Systems, Minneapolis, MN, USA) following the manufacturer's instructions. Briefly, each control and sample was placed in two wells, and standards were added to each well; the wells were incubated for 2 h at room temperature (15°C–25°C), followed by aspiration and washing of each well four times. Thereafter, each well was incubated with Mouse SDF‐1α conjugate for 2 h and incubated with the substrate solution for 30 min, both at room temperature (15°C–25°C). The stop solution was added to terminate the reaction, and the resulting optical density was measured at 450 nm using an ELISA microplate reader (Multiskan Sky; Thermo Fisher Scientific, Waltham, MA, USA).

### 2.6 Reverse Transcription Quantitative Real‐Time Polymerase Chain Reaction (PCR) Analysis

The moved M2 and the surrounding periodontal tissue were separated from the alveolar bone. Total RNA was isolated from the entire periodontal tissues using the PureLink FFPE Total RNA Isolation Kit (Invitrogen, Carlsbad, CA, USA). Isolated total RNA was converted into complementary DNA via reverse transcription with random primers using the PrimeScript RT Master Mix (Perfect Real‐Time) Kit (Takara Bio, Shiga, Japan). Real‐time PCR assays were performed in triplicate for each sample using a 7500 Real‐Time PCR System (Applied Biosystems, Thermo Fisher Scientific) with gene‐specific primers (Takara Bio) and TaqMan Gene Expression Assays (Applied Biosystems). The primers used for real‐time PCR amplification of the target genes were as follows: bone gamma‐carboxyglutamate protein (BGLAP) (Rn00566386 g1), RUNX2 (Rn01512298 m1), SDF‐1 (Rn00573260 m1), CXCR4 (Rn00573522 s1), and glyceraldehyde‐3‐phosphate dehydrogenase (GAPDH) (Rn01775763 g1). The expression levels of each target gene were standardized with reference to the GAPDH mRNA expression level.

### 2.7 Statistical Analysis

GraphPad Prism 9 (GraphPad Software Inc., San Diego, CA, USA) was used for statistical analysis. The results are presented as the mean ± standard deviation. After normality testing using the Shapiro–Wilk test to examine the distribution of all data, an unpaired Student's t‐test was used for between‐group comparisons, and Spearman's correlation coefficient was used for correlation analysis between tooth movement and root inclination angle, with *p* < 0.05, indicating significant differences.

## 3 Results

### 3.1 Body Weight

**Figure 1:**
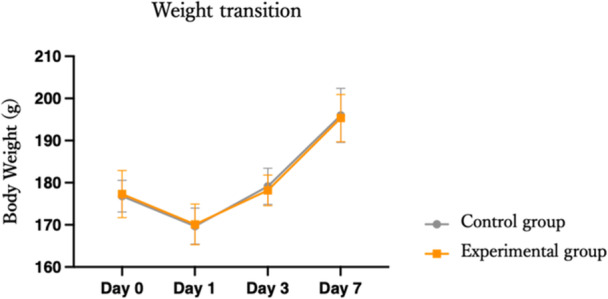
Longitudinal effects of AMD3100 on body weight. AMD3100 did not affect body‐weight changes in rats on Days 1, 3, and 7 of the experiment.

Rat body weights were recorded on experimental Days 1, 3, and 7. No significant differences were observed in body weight between the control and experimental groups throughout the experimental period (Figure 1).

### 3.2 OTM

**Figure 2:**
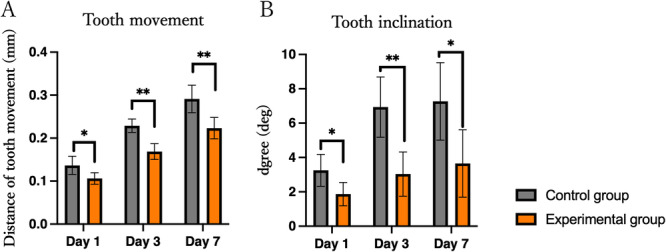
Longitudinal effects of AMD3100 on tooth movement and tooth inclination angle. AMD3100 reduced both tooth movement and mesial inclination angle. (A) M2 displacement was evaluated from micro‐CT images on Days 1, 3, and 7 after the start of the experiment in control and experimental groups. (B) The mesial inclination angle of the mesial root of M2 was measured from micro‐CT images on the same days. *p < 0.05, **p < 0.01.

**Figure 3:**
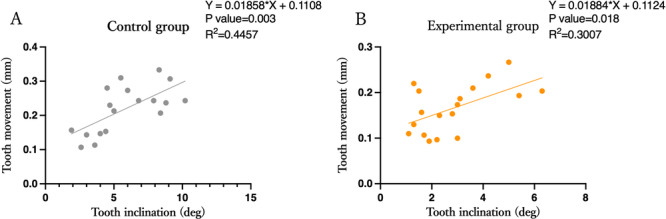
Correlation between tooth inclination angle and tooth movement distance. Both control and experimental groups showed a positive correlation between movement distance and inclination angle. (A) In the control group, the correlation between M2 displacement and mesial‐root inclination angle was *r* = 0.45, **p < 0.01. (B) In the experimental group, the correlation was *r* = 0.30, *p < 0.05.

The amount of tooth movement was significantly reduced in the experimental group compared with the control group after Day 1 (0.1061 ± 0.0132 mm vs. 0.1367 ± 0.0213 mm, *p* = 0.014), Day 3 (0.1733 ± 0.0246 mm vs. 0.2289 ± 0.0156 mm, *p* = 0.009), and Day 7 (0.2189 ± 0.0296 mm vs. 0.2911 ± 0.0320 mm, *p* = 0.004). Similarly, the M2 proximal root inclination was significantly smaller in the experimental group than in the control group after Day 1 (1.867 ± 0.683° vs. 3.250 ± 0.929°, *p* = 0.015), Day 3 (3.033 ± 1.285° vs. 6.933 ± 1.750°, *p* = 0.001), and Day 7 (3.650 ± 1.964° vs. 7.267 ± 2.254°, *p* = 0.014) of OTM. (Figure 2) Both the control (*r* = 0.668, *p* = 0.003) and experimental (*r* = 0.548, *p* = 0.018) groups showed a significant positive correlation between changes in M2 proximal root inclination and M2 tooth movement (Figure 3).

### 3.3 Micro‐CT Analysis

**Figure 4:**
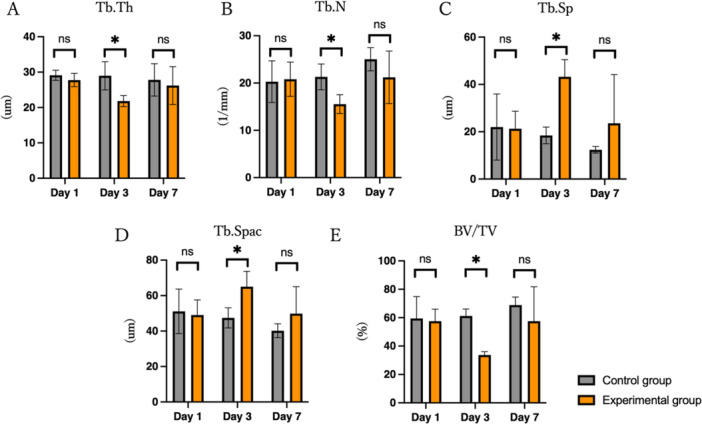
Longitudinal effects of AMD3100 on bone structure parameters. On Day 3, AMD3100 increased trabecular separation and spacing in the experimental group while decreasing trabecular thickness, trabecular number, and bone‐volume fraction. Three‐dimensional image‐analysis software was used to evaluate the microarchitecture of the alveolar bone. (A) Tb.Th, trabecular thickness; (B) Tb.N, trabecular number; (C) Tb.Sp, trabecular separation; (D) Tb.Spac, trabecular spacing; (E) BV/TV, bone‐volume/tissue‐volume ratio. *p < 0.05; ns, not significant.

On Day 3, the experimental group showed a significant increase in trabecular separation (Tb.Sp: 43.24 ± 7.24 μm vs. 18.45 ± 3.53 μm, *p* = 0.006) and spacing (Tb.Spac: 65.06 ± 8.64 μm vs. 47.42 ± 5.68 μm, *p* = 0.042) compared with those observed in the control group, as shown by alveolar bone analysis. Nevertheless, trabecular thickness (Tb.Th: 21.82 ± 1.56 μm vs. 28.97 ± 3.97 μm, *p* = 0.044), trabecular number (Tb.N: 15.55 ± 1.98/mm vs. 21.31 ± 2.73/mm, *p* = 0.042), and bone volume fraction (BV/TV: 33.73 ± 2.30% vs. 61.17 ± 4.96%, *p* = 0.001) significantly decreased in the experimental group on Day 3. No significant differences were noted in any parameter between the experimental and control groups on Days 1 and 7 (Figure 4).

### 3.4 Histological and Immunohistochemical Observations

**Figure 5:**
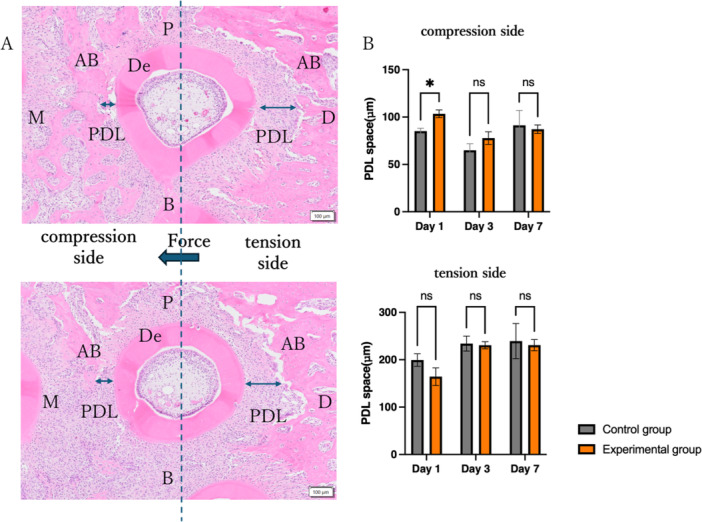
Effect of AMD3100 on early periodontal ligament remodeling during orthodontic tooth movement. (A) Representative hematoxylin and eosin‐stained horizontal sections of the distopalatal root of the maxillary second molar (M2) on Day 1 of mesial tooth movement. The upper panel shows the control group, and the lower panel shows the AMD3100‐treated group. (B) Changes in periodontal ligament (PDL) width on the compression and tension sides. AMD3100 administration increased the PDL space on the compression side during the initial phase of tooth movement, whereas no significant difference was observed on the tension side. Scale bar = 100 µm. AB, alveolar bone; B, buccal; D, distal; De, dentin; M, mesial; P, palatal; PDL, periodontal ligament.

In the HE‐stained sections, the space of the PDL decreased on the M2 compression side and increased on the tension side in both groups. Moreover, on the compression side, the PDL fibers showed irregular alignment with narrowing of the vascular lumens, and the alveolar bone surface appeared uneven. In contrast, on the tension side, the PDL space was widened, the fibers exhibited relatively regular alignment, and dilatation of the vascular lumens was observed. Furthermore, bone remodeling in the M2 inter‐root septum decreased (Figure 5A).

Quantitative analysis of the PDL width revealed a significantly greater PDL width on the compression side in the experimental group than in the control group on Day 1 (*p* = 0.048). No significant differences were observed on Days 3 or 7. Similarly, no significant differences in PDL width were detected on the tension side at any time point (Figure 5B).

**Figure 6:**
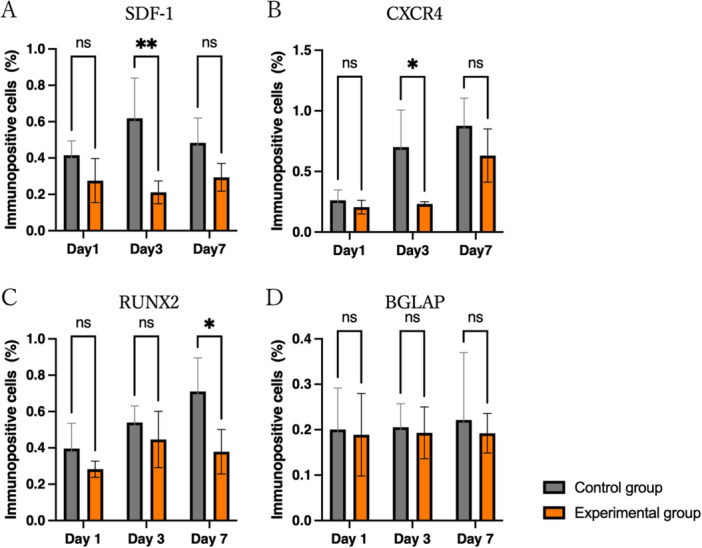
Longitudinal effect of AMD3100 on the percentages of SDF‐1‐, CXCR4‐, RUNX2‐, and BGLAP‐positive cells on the tension side of M2. (A–D) Quantitative analysis of the percentages of SDF‐1‐, CXCR4‐, RUNX2‐, and BGLAP‐positive cells in the tension side of the M2 periodontal ligament on Days 1, 3, and 7 after the start of the experiment by immunohistochemical staining. ns, not significant. *p < 0.05, **p < 0.01.

Immunohistochemical analysis demonstrated that the percentages of SDF‐1‐positive cells (*p* = 0.007) and CXCR4‐positive cells (*p* = 0.027) were significantly lower in the experimental group than in the control group on Day 3. In addition, the percentage of RUNX2‐positive cells was significantly decreased in the experimental group on Day 7 (*p* = 0.027). In contrast, no significant differences in the percentage of BGLAP‐positive cells were observed between the groups at any time point (Figures 6 and S4A–D).

### 3.5 SDF‐1 Concentration

**Figure 7:**
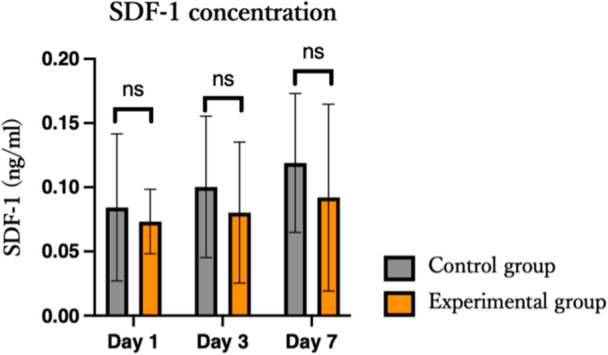
Longitudinal effect of AMD3100 on peripheral blood SDF‐1 concentrations. Peripheral‐blood SDF‐1 levels were measured by ELISA on days 1, 3, and 7 of the experiment. ns, not significant.

ELISA results for SDF‐1 levels in peripheral blood samples showed no significant differences between the control and experimental groups on Days 1, 3, and 7 (Figure 7). On Day 1, the mean concentration was 0.084 ± 0.057 ng/ml in the control group and 0.073 ± 0.025 ng/ml in the experimental group (*p* = 0.78). On Day 3, the concentrations were 0.100 ± 0.055 and 0.080 ± 0.055 ng/ml in the control and experimental groups, respectively (*p* = 0.68). On Day 7, the control group showed 0.119 ± 0.054 ng/ml, while the experimental group showed 0.092 ± 0.073 ng/ml (*p* = 0.63).

### 3.6 mRNA Expression

**Figure 8:**
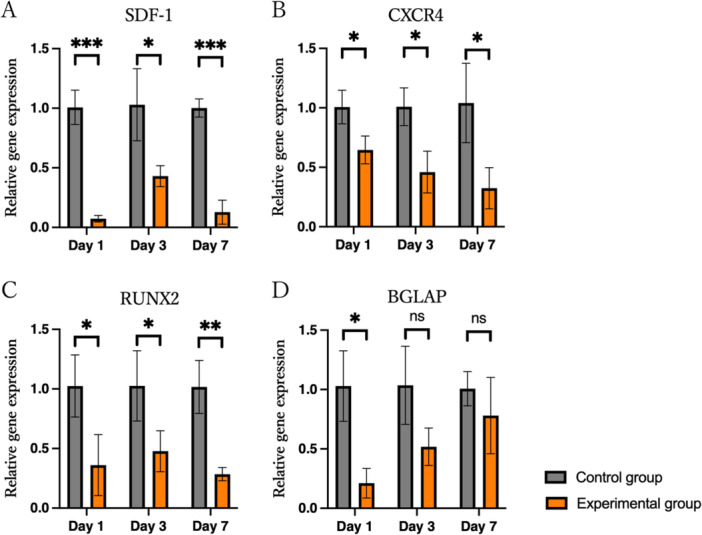
Longitudinal effect of AMD3100 on the expression levels of SDF‐1, CXCR4, and RUNX2. (A–D) On Days 1, 3, and 7 after the start of the experiment, the expression of SDF‐1, CXCR4, RUNX2, and BGLAP in M2 and the surrounding alveolar bone was quantified by qRT‐PCR. ns, not significant. *p < 0.05, **p < 0.01, ***p < 0.001.

SDF‐1, CXCR4, and RUNX2 expression levels significantly decreased on Days 1, 3, and 7 in the experimental group. For SDF‐1, the mean expression levels were 1.007 ± 0.145 versus 0.073 ± 0.027 on Day 1 (*p* < 0.001), 1.029 ± 0.302 versus 0.430 ± 0.088 on Day 3 (*p* = 0.030), and 1.002 ± 0.076 versus 0.168 ± 0.033 on Day 7 (*p* < 0.001). CXCR4 expression was also significantly lower in the experimental group, with values of 1.007 ± 0.141 versus 0.646 ± 0.117 on Day 1 (*p* = 0.027), 1.009 ± 0.159 versus 0.460 ± 0.176 on Day 3 (*p* = 0.016), and 1.041 ± 0.334 versus 0.324 ± 0.173 on Day 7 (*p* = 0.030). Similarly, RUNX2 expression decreased significantly in the experimental group at all time points: 1.025 ± 0.261 versus 0.361 ± 0.255 on Day 1 (*p* = 0.035), 1.026 ± 0.295 versus 0.478 ± 0.172 on Day 3 (*p* = 0.049), and 1.017 ± 0.223 versus 0.285 ± 0.055 on Day 7 (*p* = 0.005). Meanwhile, the BGLAP expression levels significantly decreased on Day 1:1.029 ± 0.297 versus 0.211 ± 0.124 (*p* = 0.012), in the experimental group; however, the decrease was not significant following Day 3: 1.036 ± 0.328 versus 0.518 ± 0.157 (*p* = 0.070) and Day 7: 1.007 ± 0.144 versus 0.780 ± 0.322 (*p* = 0.33) (Figure 8).

## 4 Discussion

In this study, we locally administered AMD3100, a competitive CXCR4 antagonist, to inhibit the SDF‐1/CXCR4 axis during OTM of the maxillary M2, thereby investigating the relationship between SDF‐1 and the osteogenic system. The OTM model involving M1 extraction followed by mesial movement of M2 induces enhanced bone remodeling through the Regional Acceleratory Phenomenon (RAP). RAP is characterized by accelerated local bone turnover, decreased bone density, and increased OTM velocity (Chen et al. 2016). It typically begins within several days after the surgical procedure and peaks at 1‐2 months (Hu and Li 2022). In our previous study, we demonstrated that SDF‐1 expression significantly increases from Day 1 after tooth extraction and plays a critical role in RAP‐mediated OTM acceleration (Rintanalert et al. 2024). Given that the SDF‐1/CXCR4 axis is highly activated during bone injury and tissue repair processes, we selected this experimental model to evaluate the biological role of the SDF‐1/CXCR4 axis under conditions of accelerated bone remodeling and tissue repair. Furthermore, extraction‐based orthodontic treatment is widely performed in clinical practice and often requires substantial tooth movement. Therefore, elucidating the mechanisms underlying accelerated tooth movement into extraction spaces is of considerable clinical importance.

Inhibition of the SDF‐1/CXCR4 axis reduced bone remodeling in the M2 interradicular septum and decreased the expression of osteoblast markers. These findings indicate attenuated differentiation and maturation of osteoblasts in the surrounding alveolar bone and suggest the involvement of the SDF‑1/CXCR4 axis in the biological responses of the bone‑forming system to orthodontic force. Histological analysis revealed that the PDL width on the compression side was significantly greater in the experimental group on Day 1, whereas no significant differences in PDL width were observed between the groups on either the compression or tension sides thereafter. In the early phase of orthodontic tooth movement, compression of the PDL is known to induce rapid recruitment of inflammatory cells, vascular remodeling, and osteoclast activation, resulting in narrowing of the PDL space (Nakamura et al. 2008). The increased PDL width observed following AMD3100 administration suggests that inhibition of the SDF‐1/CXCR4 axis attenuated these early biological responses on the compression side and delayed tissue remodeling within the compressed PDL. However, the effect appeared to be very short‐term, and the absence of changes in PDL width on the tension side indicates that not only biological responses but also alterations in the tooth axis and subsequent changes in the alveolar bone support may have contributed to the findings. These results should therefore be interpreted as the outcome of multiple interacting factors. Both the amount of tooth movement and the mesial tilt angle of M2 were reduced, consistent with previous findings (Hatano et al. 2018). OTM occurs through the activation of alveolar bone remodeling in the periodontal tissues induced by orthodontic force, involving osteoclasts, osteoblasts, and osteocytes (Okada et al. 2016).

The reduced tooth movement is thought to stem primarily from a decrease in osteoclast number and activity caused by inhibition of the SDF‐1/CXCR4 axis.

An important aspect of this study is that it suggests the possible involvement of the SDF‐1/CXCR4 axis not only in osteoclast‐mediated bone resorption but also in osteoblast‐related bone formation under orthodontic force. Transient inhibition of SDF‐1/CXCR4 signaling reduced the amount of tooth movement and induced statistically significant changes in alveolar bone parameters within the interradicular septum only on Day 3 after OTM. At the gene expression level, SDF‐1, CXCR4, and RUNX2 were consistently downregulated from Day 1 through Day 7, whereas BGLAP expression was decreased only on Day 1. These time‐dependent molecular changes were partly supported by the immunohistochemical findings on the tension side of the PDL. The percentages of SDF‐1‐positive and CXCR4‐positive cells were significantly lower on Day 3, and the percentage of RUNX2‐positive cells was reduced on Day 7 in the experimental group. In contrast, no significant differences were observed in the percentage of BGLAP‐positive cells at any time point.

Previous studies have reported that SDF‐1/CXCR4 signaling is involved in osteogenesis in the temporomandibular joint (Yang et al. 2020), and that disruption of this signaling suppresses osteogenic differentiation in mesenchymal stem cells through downregulation of the RUNX2 pathway (Li et al. 2017). In the present study, the reduction in RUNX2 expression and RUNX2‐positive cells suggests that inhibition of the SDF‐1/CXCR4 axis may attenuate osteoblast differentiation on the tension side of the PDL during OTM. Because RUNX2 is a key transcription factor involved in osteoblast differentiation and contributes to the expression of BGLAP, a marker of mature osteoblasts, reduced RUNX2 activity may have led to the transient decrease in BGLAP gene expression observed on Day 1.

However, the discrepancy between BGLAP gene expression and BGLAP immunostaining should be interpreted carefully. Although BGLAP mRNA expression was reduced on Day 1, the percentage of BGLAP‐positive cells on the tension side of the PDL was not significantly altered. This may indicate that transient inhibition of SDF‐1/CXCR4 signaling primarily affects early osteoblast differentiation rather than the maintenance of mature osteoblasts. In other words, during orthodontic tooth movement under tooth‐extraction‐induced RAP, bone formation on the tension side may be delayed rather than completely suppressed. RUNX2, which reflects an earlier stage of osteoblast differentiation, may therefore have been more sensitive to the early changes examined in this study, whereas BGLAP, which reflects a more mature osteoblast phenotype, may not have shown detectable histological changes within the same observation period.

It is also possible that RUNX2‐independent osteogenic pathways contributed to the maintenance of BGLAP expression. For example, Wnt signaling is known to promote osteoblast differentiation and activity (Karner and Long 2017), and both TGF‐β1 and active vitamin D have been reported to upregulate osteocalcin expression (Fukui 2010). Therefore, compensatory activation of alternative osteogenic pathways may have helped maintain BGLAP‐positive cells despite the reduction in RUNX2. Taken together, these findings suggest that the SDF‐1/CXCR4 axis may contribute to the time‐dependent regulation of both bone resorption and bone formation during the early stages of OTM, although further studies are required to clarify the precise mechanisms linking SDF‐1/CXCR4 signaling to force‐induced bone formation.

The central question is whether SDF‐1 regulates BGLAP expression “directly” or “indirectly.” To date, no reports have demonstrated a direct effect of SDF‐1 on BGLAP transcription. However, several studies have shown that cells recruited by elevated SDF‐1 influence BGLAP expression. For example, factors secreted by recruited osteoclast precursors, such as platelet‐derived growth factor‐BB and semaphorin 4D, suppress osteoblast differentiation (Kubota et al. 2002; Han et al. 2018). Likewise, TNF‐α and IL‐1β released by recruited immune cells inhibit osteoblast differentiation (Kim et al. 2002), and migrated BMSCs promote osteoblast differentiation (Li et al. 2017). These findings suggest that SDF‐1 does not act directly on osteoblasts; instead, it modulates the cytokine network through surrounding cells, such as osteoclasts, immune cells, and BMSCs, leading to secondary inhibition of osteoblast differentiation.

We performed a bone‐morphometric analysis focusing on bone volume fraction(BV/TV), Tb.Th, Tb.N and Tb.Sp. Previous studies on OTM have reported that OTM decreases bone volume and tissue density, increases trabecular spacing and thickness (Chang et al. 2019), and, when the SDF‐1/CXCR4 axis is blocked, further increases Tb.Th and Tb.Sp while reducing Tb.N (Ongprakobkul et al. 2022). In our experiment, significant differences in bone parameters were observed only on Day 3 after the initiation of force application. Specifically, short‐term blockade of the SDF‐1/CXCR4 axis led to increased Tb.Sp and trabecular spacing and decreased Tb.Th, Tb.N, and BV/TV. A prior study showed that bone resorption peaks 48–72 h after mechanical loading, followed by a compensatory phase of bone formation (An et al. 2017). Our findings are consistent with this timeline, confirming that transient disruption of SDF‐1/CXCR4 signaling markedly impairs local bone remodeling. The temporal pattern of remodeling observed herein reflects the biological dynamics induced by corrective forces. Although downregulation of SDF‐1, CXCR4, and RUNX2 was evident on D 1 post‐loading, no significant structural changes were detected, likely because of the lag between gene‐expression alterations at the tissue level and the emergence of phenotypic changes. By Day 7, the structural differences had diminished. In addition to the SDF‐1/CXCR4 axis, several other signaling pathways, such as the Vascular Endothelial Growth Factor (Clarkin and Gerstenfeld 2013) BMP (Zhao et al. 2019), and Wnt pathways (Westendorf et al. 2004) are involved in bone formation. Adaptive or compensatory activation of these pathways may partially restore bone formation in response to orthodontic forces via alternative mechanisms. These results indicate that the SDF‐1/CXCR4 signaling pathway plays a time‐dependent role in regulating the balance between bone resorption and formation, particularly during the early phase of OTM.

In this study, we focused on SDF‐1, which is constitutively expressed in periodontal tissues. SDF‐1 has been reported to play a central role in the differentiation and function of osteoblasts and osteocytes, as well as in bone remodeling and formation. Specifically, SDF‐1 promotes the recruitment, differentiation, and maturation of bone‐marrow mesenchymal stem cells and osteoblast precursors, and it is essential for maintaining and repairing bone‐tissue homeostasis. CXCR4‐positive stem cells can differentiate into osteoblasts, chondrocytes, and vascular endothelial cells required for bone formation and repair, and SDF‐1 facilitates the osteogenic process by regulating the proper localization of stem cells within the bone marrow (Zhu et al. 2011; Cristino et al. 2008). However, the impact of SDF‐1 expression on bone formation on the tension side of the PDL in response to orthodontic force remains unclear. The results of this study suggest that SDF‐1 plays an important role in bone formation on the tension side during OTM.

Moreover, SDF‐1 is involved in the maintenance and guidance of stem and progenitor cells within the bone marrow (Meng et al. 2021). Recent studies have also shown that SDF‐1 participates in the migration, differentiation, and regenerative processes of human stem cells derived from the apical papilla (SCAP) (Nagata et al. 2025). These findings indicate that SDF‐1 contributes to various dental functions. Our present results demonstrate that SDF‐1 plays a crucial role in load‐induced bone formation during OTM.

Despite AMD3100's short half‐life of several hours (De Clercq 2009), a single administration of AMD3100 decreased SDF‐1 expression throughout the experimental period. SDF‐1 expression has been reported to increase on the compression side of the PDL from Day 1, peak on Day 3, and subside by Day 7 (Nakamura et al. 2008). Conversely, although SDF‐1 is rapidly expressed on the tension side of the PDL, its temporal pattern remains unclear. SDF‐1 is produced by osteocytes and osteoblasts in response to mechanical stimulation and promotes osteoblast differentiation and activity via paracrine signaling (Chang et al. 2020). The present results suggest that residual AMD3100 in the local tissue may have suppressed the up‐regulation mechanisms of SDF‐1, sustaining its inhibitory effect until Day 3, when expression would normally peak. Consequently, RUNX2, whose expression is directly activated by SDF‐1, likely followed a similar pattern. Furthermore, previous reports indicate that AMD3100 suppresses inflammatory cytokines (Huang et al. 2013), and this suppression of inflammatory cytokines leads to reduced SDF‐1 expression (Jung et al. 2006). It is conceivable that the early suppression of inflammatory cytokines may have subsequently influenced the subsequent decrease in SDF‐1 expression.

Our findings suggest that the SDF‐1/CXCR4 axis not only participates in osteoclast mobilization but also influences early osteoblast activity. The signaling pathways governing bone remodeling facilitate the regulation of the balance between bone resorption and formation. This knowledge may contribute to safer, more predictable orthodontic outcomes in clinical practice, such as controlling tooth‐movement speed, reducing the risk of root resorption, and enhancing post‐treatment stability.

This study has several limitations. First, although AMD3100 was used to inhibit the SDF‑1/CXCR4 axis, rather than an anti–SDF‑1 antibody, AMD3100—despite being a widely used CXCR4 antagonist—may still exert off‑target effects. Therefore, the observed outcomes cannot completely exclude the possibility of non‑specific actions. Second, the mechanism by which inhibition of the SDF‑1/CXCR4 axis leads to reduced SDF‑1 expression remains unclear. Although previous studies have reported findings consistent with our results, the underlying mechanism responsible for the decreased expression of SDF‑1 was not elucidated in the present study. To clarify the specific role of AMD3100, further investigations employing more detailed genetic approaches will be necessary. Third, the gene expression analysis in this study was performed using the entire periodontal tissue surrounding M2, excluding the M2 roots. To more precisely examine the role of the SDF‑1/CXCR4 axis in the bone‑forming system during orthodontic tooth movement, gene expression analysis limited specifically to the tension side of the PDL would have been ideal. This limitation has likely contributed to the discrepancies observed between the gene expression data and the immunohistochemical findings.

## 5 Conclusions

This study demonstrated that local inhibition of the SDF‑1/CXCR4 signaling reduced OTM and altered alveolar bone remodeling after tooth extraction. The combined gene expression and immunohistochemical findings suggest that the SDF‐1/CXCR4 axis may contribute to osteoblast differentiation, particularly through RUNX2‐related responses, on the tension side of the PDL during the early phase of OTM. Further elucidation of the underlying biological mechanisms of the effects of SDF‑1 on OTM will contribute to the development of novel strategies for precise orthodontic control.

## Author Contributions

Conceptualization: Yuji Ishida, Yoshiyuki Hamada, and Albert Chun‐Shuo Huang. Methodology: Albert Chun‐Shuo Huang, Duangtawan Rintanalert, Kai Li, and Kenta HamaharaS. Validation: Albert Chun‐Shuo Huang, Duangtawan Rintanalert, Jun Hosomichi, and Risa Usumi‐Fujita. Formal analysis: Yoshiyuki Hamada. Investigation: Yoshiyuki Hamada, Albert Chun‐Shuo Huang, and Kenta Hamahara. Resources: Jun Hosomichi and Risa Usumi‐Fujita. Data curation: Yoshiyuki Hamada and Albert Chun‐Shuo Huang. Writing—original draft preparation: Yoshiyuki Hamada. Writing—review and editing: Yuji Ishida and Takashi Ono. Funding acquisition: Yuji Ishida. Supervision: Yuji Ishida and Takashi Ono. Project administration: Yuji Ishida. All authors have read and agreed to the published version of the manuscript.

## Ethics Statement

All animal experiments were approved by the Institutional Animal Care and Use Committee of the Institute of Science Tokyo (approval number 2021115C) and complied with the ARRIVE guidelines.

## Consent

The authors have nothing to report.

## Conflicts of Interest

The authors declare no conflicts of interest.

## Permission to Reproduce Material From Other Sources

The authors have nothing to report.

## Supporting information


**Figure S1:** Schematic illustration of the experimental animal model and orthodontic tooth movement procedure. The maxillary first molar (M1) was extracted in rats, and the maxillary left second molar (M2) was moved mesially using a Ni‐Ti coil spring placed between the maxillary incisors and M2. The arrow indicates the direction of tooth movement. Red circles indicate the sites of local drug injection.

**Figure S2:** Schematic illustration of micro‐CT measurement of mesial movement of M2. A reference line was established on the contralateral non‐moved control side, passing through the interdental space between M2 and M3 and perpendicular to the palatal midline suture. The distance from this reference line to the distal surface of M2 on the experimental side was measured as the amount of orthodontic movement of M2. The green line indicates the reference line, the red line indicates the distal surface of M2 on the experimental side, and the blue double‐headed arrow indicates the measured tooth movement distance.

**Figure S3:** Representative micro‐CT images showing the region of interest for bone analysis. Representative micro‐CT images after orthodontic movement of M2 are shown. The yellow‐highlighted area indicates the region of interest (ROI) in the interradicular septum of M2 used for bone analysis.

**Figure S4:** Immunohistochemical staining of the tension side of M2. (A–D) Representative immunohistochemical images of SDF‐1 (A), CXCR4 (B), RUNX2 (C), and BGLAP (D) in the tension side of M2. Images were obtained at ×400 magnification from the distal surface of the distopalatal root of M2. Three non‐overlapping regions of interest (80 × 80 μm) were analyzed using ImageJ software. Positive cells were quantified as the percentage of immunopositive cells within the selected ROIs. Black arrows indicate stained cells. Scale bar: 20 µm.

## Data Availability

Data supporting the findings of this study are available from the corresponding author upon reasonable request.
