# Supplementary material for: Effects of Stromal Cell‐Derived Factor 1 on Osteoblast Activity During Orthodontic Tooth Movement
Source: Clin Exp Dent Res. 2026 Aug 13;12(4):e70425. doi: 10.1002/cre2.70425 (PMC13472516; doi:10.1002/cre2.70425)
Supplement: Supplementary file 1 [file CRE2-12-e70425-s003.docx]

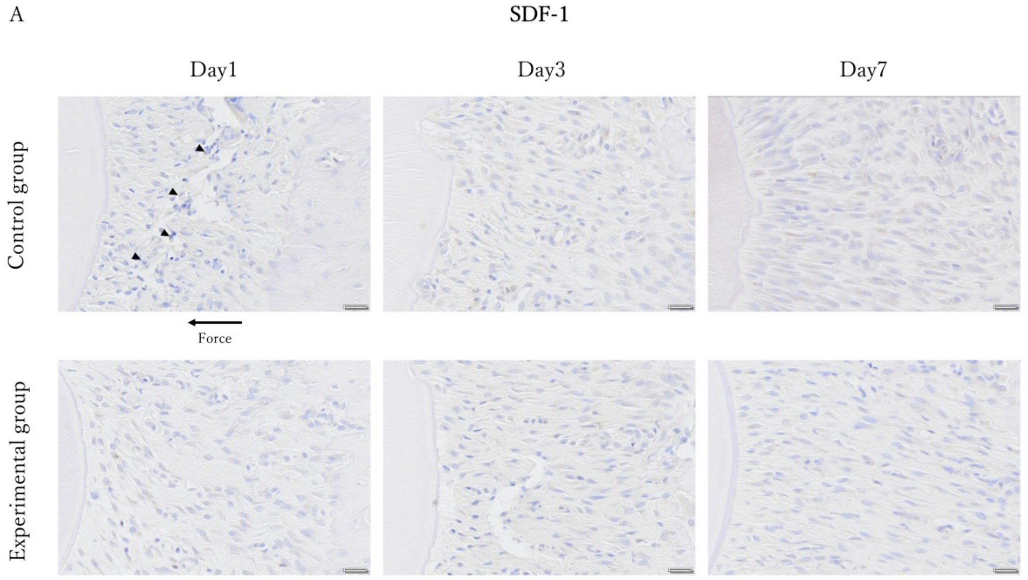
A
SDF-1
Day1
Day3
Day7
Control group
Experimental group
Force

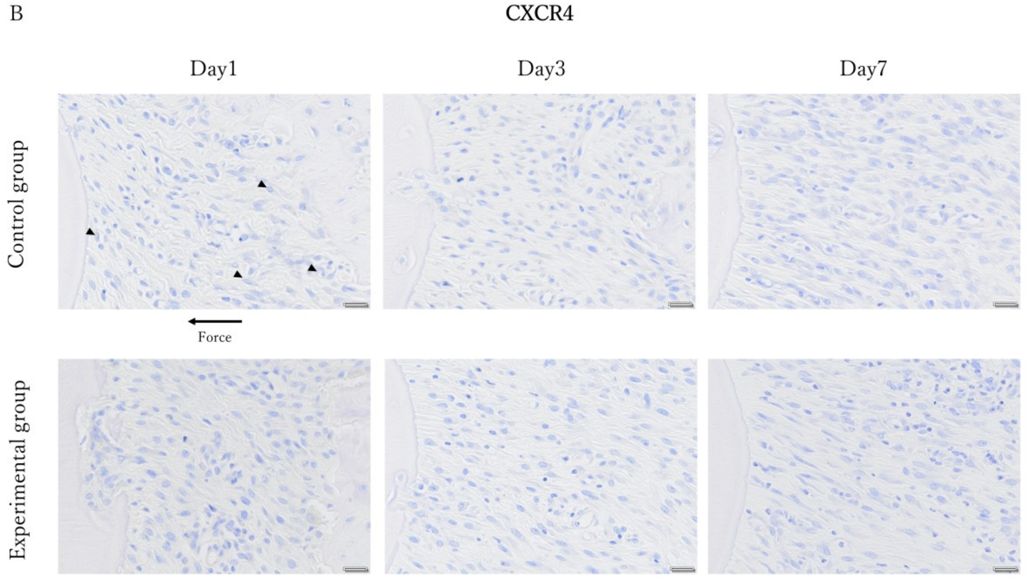
B
CXCR4
Day1
Day3
Day7
Control group
Experimental group
Force

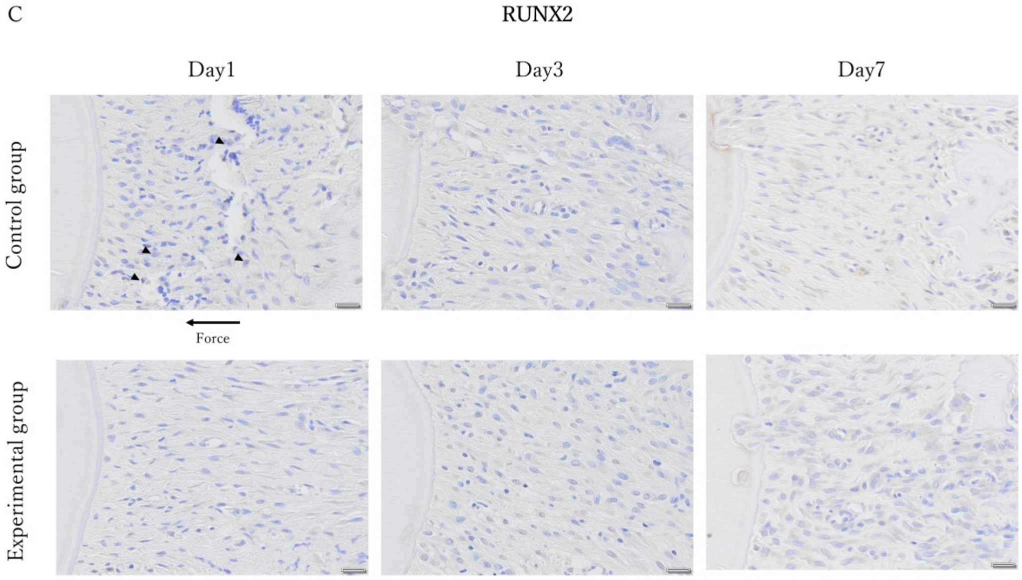
C
RUNX2
Day1
Day3
Day7
Control group
Force
Experimental group

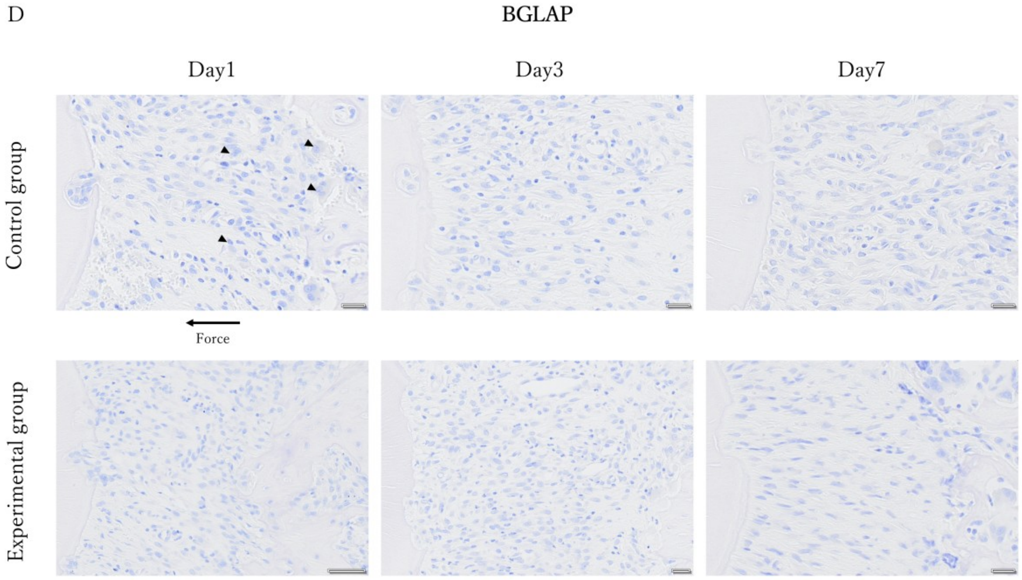
D
BGLAP
Day1
Day3
Day7
Control group
Force
Experimental group
